# An Inexpensive Staining Alternative for Gelatin Zymography Gels

**DOI:** 10.3390/mps2030061

**Published:** 2019-07-24

**Authors:** Christian Wechselberger, Christian Doppler, David Bernhard

**Affiliations:** Center for Medical Research, Medical Faculty, Johannes Kepler University, 4020 Linz, Austria

**Keywords:** gelatin zymography, Coomassie, Ponceau, inexpensive staining method

## Abstract

Zymography is a widely used electrophoretic method to determine proteolytic activities in samples from various sources. The method is based on copolymerizing a suitable protein substrate within a sodium dodecyl sulfate-polyacrylamide gel. Following electrophoretic separation of the protease containing samples and a suitable incubation period, degradation of the substrate can be visualized through staining with Coomassie blue. Sites of proteolysis become visible as white bands on a dark blue background. However, this staining protocol requires considerable amounts of ethanol and acetic acid to remove unbound dye molecules. In this report, we describe a new staining protocol using Ponceau S which offers substantial advantages in terms of assay usability and cost reduction, especially when performing large quantities of zymograms or in resource-limited settings. Fast and reproducible staining of zymograms with our protocol is demonstrated, and reliable quantitation of proteolytic activity in comparison to the standard Coomassie staining procedure is shown.

## 1. Introduction

Matrix metalloproteinases (MMPs) are Ca^2+^/Zn^2+^ dependent endopeptidases necessary for the degradation of extracellular matrix (ECM) components during a multitude of physiological and pathophysiological processes (e.g., embryonic development, aging, tissue remodeling, tumor growth, and invasion) [1,2,3]. The gelatinases MMP-2 (gelatinase A) and MMP-9 (gelatinase B) are two members of the MMP family that have been extensively studied in this respect. Both enzymes are able to cleave a variety of proteins present in the extracellular matrix.

Zymography, among other techniques, is a well-established method used for the detection and characterization of extracellular matrix-degrading enzymes from tissue extracts, cell cultures, serum or urine to name a few examples [4,5,6,7]. Routinely SDS gels copolymerized with a suitable protein substrate (e.g., gelatin) are used and protease activities can be analyzed with this method after enzyme renaturation through SDS-removal following electrophoretic separation [8]. Additional benefits of this method include **(a)** the possibility to detect and quantify several proteases exerting activity on the same substrate in parallel, **(b)** detection of the proteolytically inactive pro-forms of proteinases since these become activated during the renaturation procedure, and **(c)** removal of tissue inhibitors of metalloproteinases (TIMPs) during electrophoresis allows for the detection of the total enzymatic activity in a given sample.

The standard staining procedure for protein gels with Coomassie is well established and remained largely unchanged for almost 30 years [9,10,11]. Although this is a very sensitive and robust technique, it requires considerable amounts of ethanol and acetic acid during the subsequent destaining steps, a fact that can substantially contribute to the final costs for analyzing zymogram gels, especially in high throughput settings. On the other hand, staining proteins with Ponceau represents a fast and very inexpensive method that is, at present, mainly used to detect proteins on nitrocellulose membranes after transfer blotting as a control for equal loading [10]. Additional applications include a Ponceau staining procedure that is used on histological slides to demonstrate any local gelatinolytic activity [12].

Reduction of reagent costs, as well as waste minimization, has always been an important aspect in life science laboratories. Previous and also very recent work has focused on reducing, e.g., the concentration of dyes for different staining applications from zymography to western blotting as well as reducing the ethanol and acetic acid concentration in Coomassie destaining solutions [4,13]. Using the method presented in this manuscript one can roughly estimate that replacing the Coomassie staining procedure with the Ponceau staining protocol can further reduce the costs of gel destaining by more than 70% from € 6.14 to € 1.71 by using the volumes indicated in the Procedure section.

## 2. Experimental Design

The goal of this work was to perform a direct comparison of the Coomassie and the Ponceau staining method for analyzing zymogram gels in terms of usability and relative sensitivity. As samples for this proof of concept approach, we have used gelatinolytic enzymes present in fetal bovine serum samples (FBS; Gibco Life Technologies, Paisley, UK). Dilution series of FBS were prepared (see Table 1) and standard zymogram experiments were performed. For reproducibility reasons, we have always prepared sufficient gelatin-containing polyacrylamide solutions for two gels in parallel, one for staining with the standard Coomassie procedure and the other being processed using our Ponceau staining protocol. Quantification of proteolytic activities on zymograms visualized by Coomassie as well as Ponceau staining was done by densitometry scanning and computer-assisted image analysis using the ImageJ program (https://imagej.nih.gov/ij/) to analyze the MMP-2 associated signal. In order to avoid possible bias due to background subtraction over the entire width of the gels, we have selected appropriate areas below the MMP-2 signal in the same lane as the respective background area and subtracted these values from the specific signal. Quadruplicate experiments were performed for each staining procedure over a time course of about six weeks using the same staining solutions without an obvious loss in performance for neither the Coomassie nor the Ponceau staining solutions.

### 2.1. Materials

#### 2.1.1. Reagents

- Heat-inactivated fetal bovine serum (Gibco Life Technologies, Paisley, UK; # 10500)
- Prestained protein standard (New England BioLabs, Frankfurt am Main, Germany; # P7719G)
- Coomassie brilliant blue R-250 (Sigma-Aldrich, Vienna, Austria; # 27816-25G)
- Acetic acid (Sigma-Aldrich, Vienna, Austria; # 33209-1L-M)
- Ethanol (Sigma-Aldrich, Vienna, Austria; # 32221-1L-M)
- Water (Sigma-Aldrich, Vienna, Austria; # 38796-1L)
- Glycerol (Sigma-Aldrich, Vienna, Austria; # G7757-1L-M)
- Ponceau S (Sigma-Aldrich, Vienna, Austria; # P3504-10G)
- Gelatin from bovine skin, type B (Sigma-Aldrich, Vienna, Austria; # G9391)
- Triton X-100 (Sigma-Aldrich, Vienna, Austria; # T8787-100ML)
- 1 M Trizma hydrochloride buffer solution (Sigma-Aldrich, Vienna, Austria; # 93313-1L)
- 2 M zinc sulfate solution (Sigma-Aldrich, Vienna, Austria; # 83265-250ML-F)
- Sodium dodecyl sulfate solution (Sigma-Aldrich, Vienna, Austria; # 05030-500ML-F)
- 1.5 M Tris pH 8.8 (Bio-Rad Laboratories, Vienna, Austria; # 161-0798)
- 0.5 M Tris pH 6.8 (Bio-Rad Laboratories, Vienna, Austria; # 161-0799)
- 30% acrylamide/bis solution, 29:1 (Bio-Rad Laboratories, Vienna, Austria; # 161-0157)
- 4× Laemmli sample buffer (Bio-Rad Laboratories, Vienna, Austria; # 161-0747)
- 10× Tris glycine SDS electrophoresis buffer (Bio-Rad Laboratories, Vienna, Austria; # 161-0772)
- Ammonium persulfate (Bio-Rad Laboratories, Vienna, Austria; # 161-0700)
- TEMED (Bio-Rad Laboratories, Vienna, Austria; # 161-0800)
- Calcium chloride (Carl Roth GmbH, Karlsruhe, Germany; # CN93.1)

#### 2.1.2. Solutions

• Gelatin solution

Prepare a 1% gelatin solution (10 mg/mL) by dissolving 0.1 g gelatin in 10 mL of water; solubilization can be enhanced by incubation at 37 °C in a water bath; store at 4 °C for up to two weeks.

• Polyacrylamide Separation Gel (8 mL/7.5%)

1.5 M Tris pH 8.8 2 mL30% Acrylamide/Bis Solution, 29:1 2 mLDeionized water 3 mLGelatin (10 mg/mL) 1 mL10% SDS 0.08 mL10% APS 0.08 mLTEMED 0.01 mL

• Polyacrylamide Stacking Gel (2.5 mL/4%)

0.5 M Tris pH 6.8 0.625 mL30% Acrylamide/Bis Solution, 29:1 0.335 mLDeionized water 1.5 mL10% SDS 0.025 mL10% APS 0.025 mLTEMED 0.005 mL

• Gelatinase Renaturation Buffer

Triton X-100 25 mL1 M Tris-HCl, pH 7.5 50 mL1 M CaCl_2_ 5 mL2 M ZnSO_4_ 1 μLDeionized water 920 mL

• Gelatinase Reaction Buffer

Triton X-100 0.5 mL1 M Tris-HCl, pH 7.5 50 mL1 M CaCl_2_ 5 mL2 M ZnSO_4_ 1 μLDeionized water 944.5 mL

• Coomassie Gel Staining Solution

Coomassie brilliant blue R 0.3 gAcetic acid 2 mLEthanol 90 mLDeionized water 108 mL

• Coomassie Gel Destaining Solution

Acetic acid 50 mLEthanol 125 mLDeionized water 325 mL

• Ponceau Gel Staining Solution

Ponceau S 0.25 gAcetic acid 10 mLDeionized water 190 mL

• Gel Preservation Solution for Coomassie Method

Glycerol 6 mLEthanol 60 mLDeionized water 134 mL

• Gel Preservation Solution for Ponceau Method

Glycerol 6 mLDeionized water 194 mL

### 2.2. Equipment

- Centrifuge 5424R (Eppendorf Austria GmbH, Vienna, Austria; # 5404000410)
- Water bath (GFL Gesellschaft für Labortechnik mbH, Burgwedel, Germany; # 1013)
- Eppendorf Research plus Micropipettes (VWR International GmbH, Vienna, Austria; # 613-1143)
- Orbital shaker (GFL Gesellschaft für Labortechnik mbH, Burgwedel, Germany; # 3017)
- PowerPac™ HC High-Current Power Supply (Bio-Rad Laboratories, Vienna, Austria; # 164-5052)
- Mini-PROTEAN^®^ Tetra Cell (Bio-Rad Laboratories, Vienna, Austria; # 1658000)
- Gel drying frames (Sigma-Aldrich, Vienna, Austria; # Z377597)
- Vilber Lourmat E-Box gel documentation imaging system (VWR International GmbH, Vienna, Austria; # 730-1486)

## 3. Procedure

### 3.1. Sample Preparation

1. Take sample and clear by centrifugation (10,000 g/5 min at room temperature).
2. Transfer supernatant into new tube and prepare samples with loading buffer (w/o β-mercaptoethanol) according to manufacturer’s instructions.
3. Incubate samples at room temperature for 10 min; do not heat denature the samples as this would inactivate enzymatic activities of the proteases.
4. Load samples onto SDS-gelatin-polyacrylamide gel.

### 3.2. Perform Gel Electrophoresis

Run the gels at 150 V (30–50 mA and 4–8 W for 2 gels) using 1x electrophoresis buffer until the dye front reaches the bottom of the gel (~55 min).

### 3.3. Gelatinase Activation/Incubation

1. After electrophoreses, carefully remove the gel from the electrophoretic plates.
2. Place the gel in a container and wash 3 times with ~50 mL gelatinase renaturation buffer for 20 min each while agitating the container.
3. Incubate the gel with ~50 mL gelatinase reaction buffer in a container at 37 °C for 18–24 h.

### 3.4. Gel Staining

#### 3.4.1. ***Option A*** Coomassie Gel Staining and Destaining

1. Stain the gel with 20 mL Coomassie gel staining solution for 1 h at room temperature; use a shaker for even distribution of the staining solution.
2. Destain the gel with Coomassie gel destaining solution for at least 30 min until clear bands are visible; change the destaining solution at least 4 times with 25 mL each.

#### 3.4.2. ***Option B*** Ponceau Gel Staining and Destaining

1. Stain the gel with 20 mL Ponceau gel staining solution for 1 h at room temperature; use a shaker for even distribution of the staining solution.
2. Destain the gel with water for at least 30 min until clear bands are visible; change the water at least 4 times with 25 mL each.

### 3.5. Gel Scan and Quantification

1. Seal the gel in a plastic wrap.
2. Immediately scan the gel with a digital scanner (include gelatinase standards for normalization purposes if necessary).
3. Quantify the integrated density values of the gelatinase bands using NIH ImageJ or a similar software.

### 3.6. Archiving

1. Equilibrate gels with ~50 mL of the appropriate gel preservation solution for 15 min.
2. Pre-wet cellophane sheets with deionized water and assemble the gel drying frames according to manufacturer’s instructions.
3. Dry the gels overnight.

## 4. Expected Results

We have demonstrated that Ponceau S staining of zymography gels can be used as a suitable replacement for Coomassie staining yielding comparable results at a very low cost. As samples, we have applied serial dilutions of fetal bovine serum (FBS; Gibco Life Technologies, Paisley, UK) (Table 1) and analyzed the performance of the novel staining procedure in terms of reproducibility and sensitivity.

**Figure 1:**
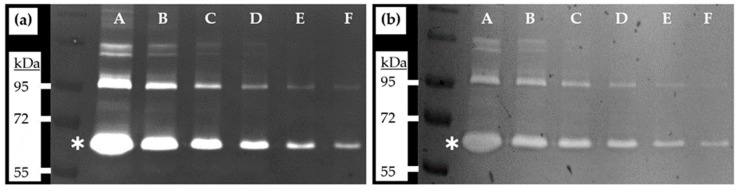
Zymography gels showing bands generated by gelatinolytic activities present in fetal bovine serum samples. (**a**) Coomassie staining; (**b**) Ponceau staining. For sample description (FBS equivalents), see Table 1. Asterisks mark the position of active MMP-2 enzymatic activity with a molecular weight of 62 kDa. Exemplary bands of the color prestained protein standard (New England Biolabs, # P7719G) are indicated.

In order to compare the performance of staining zymography gels with Ponceau in respect to the standard Coomassie procedure, we took pictures of the respective gels with a gel scanner. Exposure time was adjusted to avoid having saturated areas in the gels that would interfere with subsequent analysis. Exemplary images of zymography gels used in the course of this work are shown in Figure 1.

**Figure 2:**
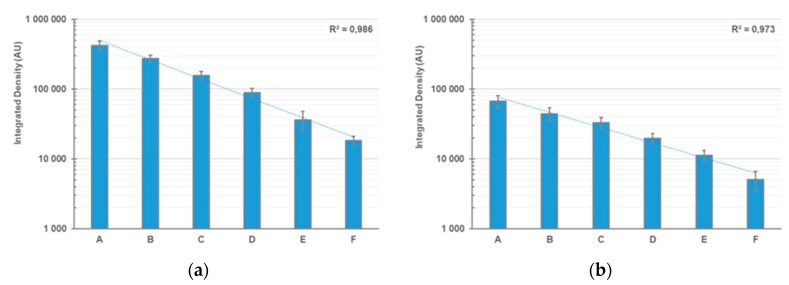
Densitometric analysis of zymography gels after using different staining methods. (**a**) Coomassie staining; (**b**) Ponceau staining. For sample description (FBS equivalents), see Table 1. Experiments were carried out as quadruplicates with four gels each for the Coomassie and Ponceau staining procedures. Data represent the mean of four independent experiments, error bars represent the respective standard deviation. Trendlines and the corresponding coefficients of determination (R squared) are shown; AU: arbitrary units of integrated density.

Measurement on gelatinolytic areas was done by using NIH ImageJ software as follows: a defined area was selected (size 100 × 50 pixels) and background values were taken next to the band of interest to avoid any bias that could be introduced due to using a global background over the whole width of the gels. This background value was then subtracted from the signal obtained by measuring the respective band in the zymogram gel and the next lane was analyzed in the same way (see Figure 2).

## 5. Conclusions

We demonstrate that the use of Ponceau staining represents an easy and inexpensive alternative detection method for the characterization of gelatinolytic activities in zymography gels. Sensitivity and linearity of the method are almost comparable to the conventional Coomassie procedure. Although there will be applications where the characterization of minute amounts of sample material from limited or expensive sources might vindicate the use of the traditional Coomassie procedure, we are convinced that Ponceau staining represents a viable alternative, given the ease of use, the reduced costs, and the concomitant reduction in chemical waste.

## Figures and Tables

**Table 1:** Serial dilutions of heat-inactivated fetal bovine serum (Gibco Life Technologies, Paisley, UK; # 10500).

Sample	FBS Equivalent
A	1 µL
B	0.5 µL
C	0.25 µL
D	0.125 µl
E	0.0625 µL
F	0.03125 µL
